# Extract of *Bletilla formosana* callus elevates cellular antioxidative activity via Nrf2/HO-1 signaling pathway and inhibits melanogenesis in zebrafish

**DOI:** 10.1186/s43141-023-00482-0

**Published:** 2023-03-06

**Authors:** Ruei-Ting Wang, Jui-Hung Yen, Yi-Chi Liao, Yi-Zhen Li, Wei-Ping Wang

**Affiliations:** 1 CHLITINA Research and Development Center, CHLITINA Holding Ltd., Taipei, 110050 Taiwan; 2 Biomedical Technology and Device Research Laboratories, Industrial Technology Research Institute, Hsinchu, 310401 Taiwan; 3 Graduate Institute of Biotechnology, National Chung-Hsing University, Taichung, 402202 Taiwan

**Keywords:** *Bletilla formosana*, Sustainable resources, Cell culture, Supercritical fluid extraction, Intracellular antioxidative activity, Melanogenesis-inhibitory effect, Zebrafish embryos

## Abstract

**Background:**

*Bletilla* species are endangered terrestrial orchids used in natural skin care formulas in Asia for a long history. In order to explore the bioactivity potential of *Bletilla* species as a cosmetic ingredient in a sustainable resource manner, the callus of *Bletilla formosana* (Hayata) Schltr. was established and extracted by an eco-friendly supercritical fluid CO_2_ extraction (SFE-CO_2_) method. The intracellular reactive oxygen species (ROS) scavenging activity and antioxidation-related gene expression of the callus extract were evaluated in both Hs68 fibroblast cells and HaCaT keratinocytes. The melanogenesis-inhibitory effect was investigated in B16F10 melanoma cells and in an in vivo zebrafish model.

**Results:**

The calli of *B. formosana* were propagated for 10–15 generations with a consistent yellow friable appearance and then subjected to SFE-CO_2_ extraction to obtain a yellow pasty extract. Obvious intracellular ROS scavenging activity of the extract was detected in both Hs68 and HaCaT cells with 64.30 ± 8.27% and 32.50 ± 4.05% reduction at the concentration of 250 μg/mL. Moreover, marked expression levels of heme oxygenase-1 (HO-1) and (NAD(P)H) quinone oxidoreductase-1 (NQO1) genes were detected after 6-h and 24-h treatments. These results indicate the cellular antioxidative activity of *B. formosana* callus extract was probably activated via the nuclear factor erythroid 2-related factor 2 (Nrf2)/HO-1 signaling pathway. Melanogenesis-inhibitory effect of the extract was observed in α-MSH stimuli-inducing B16F10 cells with 28.46% inhibition of intracellular melanin content at the concentration of 50 μg/ml. The effect was confirmed with in vivo zebrafish embryos that showed a relative pigmentation density of 80.27 ± 7.98% at the concentration of 100 μg/mL without toxicity.

**Conclusion:**

Our results shed light on a sustainable utilization of *Bletilla* species as a potential ingredient for skin.

## Background

“Bai ji,” Chinese name for tubers of *Bletilla striata*, has been used as traditional Chinese medicine (TCM) for thousands of years in the treatment of several health dysfunctions, including gastrointestinal disorders, ulcers, lung disorders, chapped skin, and traumatic bleeding [1]. More frequently, *B. striata* was historically used in the formula for skin whitening, such as qī bái sàn (七白丸) from traditional Chinese medicine books pǔ jì fang (普濟方) and qī bái tǐng zǐ gāo (七白挺子膏) from tài píng shèng huì fang (太平聖惠方) [2]. Recent pharmacological research showed that *Bletilla* species exhibit antiulcer, anti-oxidative, antimicrobial, anti-inflammatory, neuroprotective, anticancer, antiviral, immunomodulatory, anti-fibrosis, anti-aging, anti-allergic, and antipruritic activities [1, 3]. Large amounts of wild *Bletilla* species have been over-collected, leading to endangered status.

*Bletilla* is a small genus of the Orchidaceae family containing only five species in the world [3], of which *B. striata*, *Bletilla ochracea* Schltr., *Bletilla sinensis* (Rolfe) Schltr., and *Bletilla formosana* (Hayata) Schltr. were recorded in Flora of China [4, 5]. Orchidaceae family plants are commonly difficult to obtain offspring in nature due to the undernourished seeds with low germination rate; the gap between supply and demand of *Bletilla* species is ever-increasing [3, 6, 7]. Plant resources have been used as natural skincare ingredients for their multiple bioactive components. Various bioactive compounds derived from plants have been applied in anti-aging [8]. However, ingredients may be influenced by harvest season and different cultivation locations, which are the major concern for their application in the pharmaceutical and cosmetics industries [9]. Therefore, the utilization of genetic improvement and biotechnology methods to obtain higher medicinal ingredient content for sustainable and stable production are promising ways to meet the increasing market demand, especially for endangered medicinal plants [10].

A currently growing interest in the market is the products of plant cell culture-derived active cosmetic ingredients. Plant cell culture technology is a prominent approach with several advantages, such as sustainable resources, stable reproduction, controlled metabolites production, and eco-friendly, which is beneficial for the increasing trend in products of low environmental impacts [11]. Many plant cell extracts were illustrated with dermatological effects, including regulation of cell division, reconstruction of the damaged epidermis, activation of cellular DNA repair, and protection against UV radiation [10, 12, 13]. For example, *Syringa vulgaris* cells present skin anti-inflammatory and anti-aging effects [14]; *Coffea benghalensis* and *Nicotiana sylvestris *cells stimulate collagen production of fibroblasts [15, 16]; *Rhus coriaria* cells accelerate skin repair [17]; *Pyrus pyrifolia* cells promote cell proliferation of keratinocyte and fibroblast [9]; *Tiarella polyphylla* cells protect fibroblast from photoaging [18].

Callus establishment and accumulation of secondary metabolites of *B. striata* has been reported [19], but the bioactivity on the dermatological application is still unclarified. In this study, the dermatological potential of *B. formosana*, a native herbal medicine used as an alternative to *B. striata* in Taiwan was investigated. The callus from seeds of *B. formosana* under controlled conditions was established and callus extract was obtained through an eco-friendly supercritical fluid CO_2_ extraction (SFE-CO_2_) method. The antioxidative activity of the callus extract was validated by intracellular reactive oxygen species (ROS) scavenging activity in both Hs68 fibroblast cells and HaCaT keratinocytes. Melanogenesis-inhibitory effect of the extract was investigated in both B16F10 cell line and zebrafish model.

## Methods

### Callus culture and extraction

Plants of *B. formosana* were collected and molecularly identified by Industrial Technology Research Institute, Hsinchu, Taiwan. Mature capsules were collected 3–4 months after pollination, sterilized in 70% (v/v) ethanol for one minute, followed immersed in 1.5% NaOCl for 20 min, and then rinsed twice with sterile distilled water. Sterile seeds were collected from capsules and cultured on callus-inducing medium according to Gamborg et al. [20] with some modifications. Briefly, seeds were inoculated on B5 medium supplemented with 2 mg/L 6-Benzyladenine (6-BA), 1 mg/L 2,4-Dichlorophenoxyacetic acid (2,4-D), 25 g/L sucrose, and 0.7% agar at 25 °C in the dark. After 2 months, calli were transferred to a new medium for subsequent subcultures and proliferation. Subcultures were carried out at 30-day intervals. The materials used in this study were harvested from 45 days cultured calli of 10–15 generations and extracted by SFE-CO_2_ after lyophilization. The extract was dissolved in DMSO and used in the following experiments.

### Cell culture and chemicals

Human foreskin fibroblast Hs68 cells (ATCC CRL-1635, BCRC60038) were purchased from Bioresource Collection and Research Center, Hsinchu, Taiwan. Human keratinocyte HaCaT cells (CLS 300,493) were purchased from Cell Line Service GmbH (Eppelheim, Germany). HaCaT was cultured in Dulbecco’s modified Eagle’s medium (DMEM) supplemented with 2 mM sodium pyruvate, and Hs68 was cultured in DMEM. These media contain 10% fetal bovine serum and 100 units/mL antibiotics. The cells were grown at 37 °C in a 5% CO_2_ incubator. All cell culture media and reagents were of reagent grade or cell-culture grade purchased from Sigma-Aldrich (St. Louis, MO, USA) or Gibco (Thermo Fisher Scientific, Inc., Carlsbad, CA, USA).

### Cell viability

Cell viability was determined by the 3-(4,5-dimethylthiazol-2-yl)-2,5-diphenyltetrazolium bromide (MTT) assay. The experiments were conducted on 96-well plates. Hs68 cells were seeded at 5 × 10^3^ cells/well, and HaCaT cells were seeded at a concentration of 1 × 10^4^ cells/well, respectively. After overnight incubation, the medium was removed and replaced with medium containing *B. formosana* callus extract dissolved in DMSO and maintained at 37 °C with 5% CO_2_ for 24 h. One hundred microliters of 0.5 mg/mL MTT solution was added to each well and incubated at 37 °C for 3 h. MTT solution was removed, and the crystal was lysed with DMSO. The absorbance of lysate was recorded at 570 nm. The results were expressed as the relative percentage of untreated cells.

### Intracellular ROS determination

The experiments were conducted on 96-well plates according to a method previously described [21]. Hs68 and HaCaT cells were seeded in density 5 × 10^3^ cells/well and 1 × 10^4^ cells/well, respectively. ROS was measured with the 2′,7′-dichlorofluorescin diacetate (DCFH-DA), and the cell viability was measured with MTT. Briefly, the cells were treated with callus extract for 24 h, followed by 1 mM H_2_O_2_ for 30 min. The cells were washed twice with PBS and incubated with either 10 μM DCFH-DA for 30 min or 0.5 mg/mL MTT for 3 h. The fluorescence intensity was recorded with excitation at 485 nm and emission at 535 nm using a SpectraMax® iD3 microplate reader (Molecular Devices). The cell viability was measured as above mentioned. The absorbance of the untreated well was used as blank. The results were expressed as the relative percentage of control cells.

### RNA isolation and real-time quantitative PCR

The transcription levels of nuclear factor erythroid 2-related factor 2 (Nrf2), catalase (CAT), superoxide dismutase (SOD), glutathione peroxidase (GPx), heme oxygenase-1 (HO-1), and (NAD(P)H) quinone oxidoreductase-1 (NQO1) genes were measured using real-time quantitative PCR. The total RNA of Hs68 fibroblast cells and HaCaT keratinocytes were extracted by Quick-RNA Miniprep Kit, R1055 (Zymo Research) according to the operation guideline. Extracted total RNA concentration was determined by absorbance at 260 nm, and the quality of total RNA was checked by measuring the ratio of absorbance at 260/280 nm and 260/230 nm using the NanoDrop™ 2000 Spectrophotometer (Thermo Fisher Scientific). cDNA was synthesized with 500 ng of total RNA using Magic RT cDNA synthesis kit (BB-DBU-RT-100, Bio-genesis). Real-time quantitative PCR was conducted according to a method previously described [22]. The mRNA expression levels of Nrf2, CAT, SOD, GPx, HO-1, and NQO1 were determined using PowerUp™ SYBR Green Master Mix (Thermo Fisher Scientific, Inc., Carlsbad, CA, USA) and QuantStudio™ 3 real-time PCR instrument (Applied Biosystems) according to instructor protocol. The relative expression of each gene was calculated by the ΔΔCt method. Τhe Ct of each gene was normalized to the Ct of GAPDH. Fold changes (arbitrary units) were determined as 2^−ΔΔCt^. Parts of primer sequences were designed using NCBI/Primer-Blast and the sequences are listed in Table 1.

**Table 1:** Primers used for real-time quantitative PCR

Genes		Sequence (5'-3')	Accession Number	Ref
GAPDH	**Forward**	ACTTCAACAGCGACACCCAC	NM_002046.7	[23]
	**Reverse**	CCCTGTTGCTGTAGCCAAATTC		
Nrf2	**Forward**	CTGCCAACTACTCCCAGGTT	NM_006164.5	
	**Reverse**	AAGTGACTGAAACGTAGCCGA		
CAT	**Forward**	AAGGTTTGGCCTCACAAGGA	NM_001752.4	[24]
	**Reverse**	GCGGTAGGGACAGTTCACAG		
SOD	**Forward**	TGGGCAATGTGACTGCTGAC	NM_000454.5	[25]
	**Reverse**	ACCAGCCAAACGACTTCCAG		
GPx1	**Forward**	GTTTGGGCATCAGGAGAACG	NM_000581.4	
	**Reverse**	CAACATCGTTGCGACACACC		
HO-1	**Forward**	CTCCACATCCAGCTCTTTGAGG	NM_002133.3	
	**Reverse**	CGTGGGCAGAATCTTGCAC		
NQO1	**Forward**	CTGAAAGGCTGGTTTGAGCG	NM_000903.3	
	**Reverse**	TCCACTCTGAATTGGCCAGAG		

### Melanin content measurement

Melanin content was determined according to previously reported study [26] with some modifications. 100 nM of α-MSH, agonist MC1R, and pretreated B16F10 melanoma cells were incubated with either the *B. formosana* callus extract or Kojic acid for 48 h. After incubation, the cultured cells were harvested with trypsin and centrifuged at 1000 rpm for 5 min. The pellets were dissolved in 1 N NaOH and incubated at 80 °C for 2 h. The absorbance of dissolved melanin was measured at 405 nm using an Epoch™ microplate reader (Bio-Tek). All samples were normalized with their protein concentration determined with Pierece™ BCA protein assay kit, 23,227 (Thermo Fisher Scientific, Inc., Carlsbad, CA, USA).

$$\mathrm{Melanin}\;\mathrm{content}\left(\%\right)$$

$$=\left[\left(A_{\mathrm{sample}}-A_{\mathrm{sample}\;\mathrm{blank}}\right)\div\left(A_{\mathrm{control}}-A_{\mathrm{control}\;\mathrm{blank}}\right)\right]\times100\%$$

$$\mathrm{Cellular}\;\mathrm{melanin}\;\mathrm{content}\;(\%)$$

$$=\left[\mathrm{melanin}\;\mathrm{content}\;(\%)\;\mathrm{protein}\;\mathrm{concentration}\;(\%)\right]\times100\%$$

### Zebrafish embryo test

Zebrafish embryos were obtained from Association for Assessment and Accreditation of Laboratory Animal Care International (AAALAC) accredited Taiwan Zebrafish Core Facility (TZCF) at National Health Research Institutes (NHRI) of Taiwan. Zebrafish in vivo assay was performed according to the previous method [27] with some modifications. The collected synchronized zebrafish embryos were arrayed by glass dropper into a 12-well plate, six embryos per well with 2-mL embryo medium, and replaced daily. The prepared callus extract solutions were added to the E3 embryo medium from 9 to 72 h post-fertilization (hpf). The positive control was 10 mM Kojic acid. Phenotype-based evaluations of body pigmentation ware performed at 72 hpf. Stereomicroscope was employed for observing the effects on the pigmentation of zebrafish. The embryos were anesthetized in tricaine methanesulfonate solution and photographed under MSV269. Image capturing and pixel measurement analysis were carried out by ImageJ software to assess the effects of callus extract on the pigmentation of zebrafish.

### Statistical analysis

All values were expressed as mean ± SD. The statistical significance of the differences between the two sample populations was determined by an unpaired two-tailed Student’s *t*-test.

## Results

### Induction, growth, and extraction of B. *formosana* callus

**Fig. 1:**
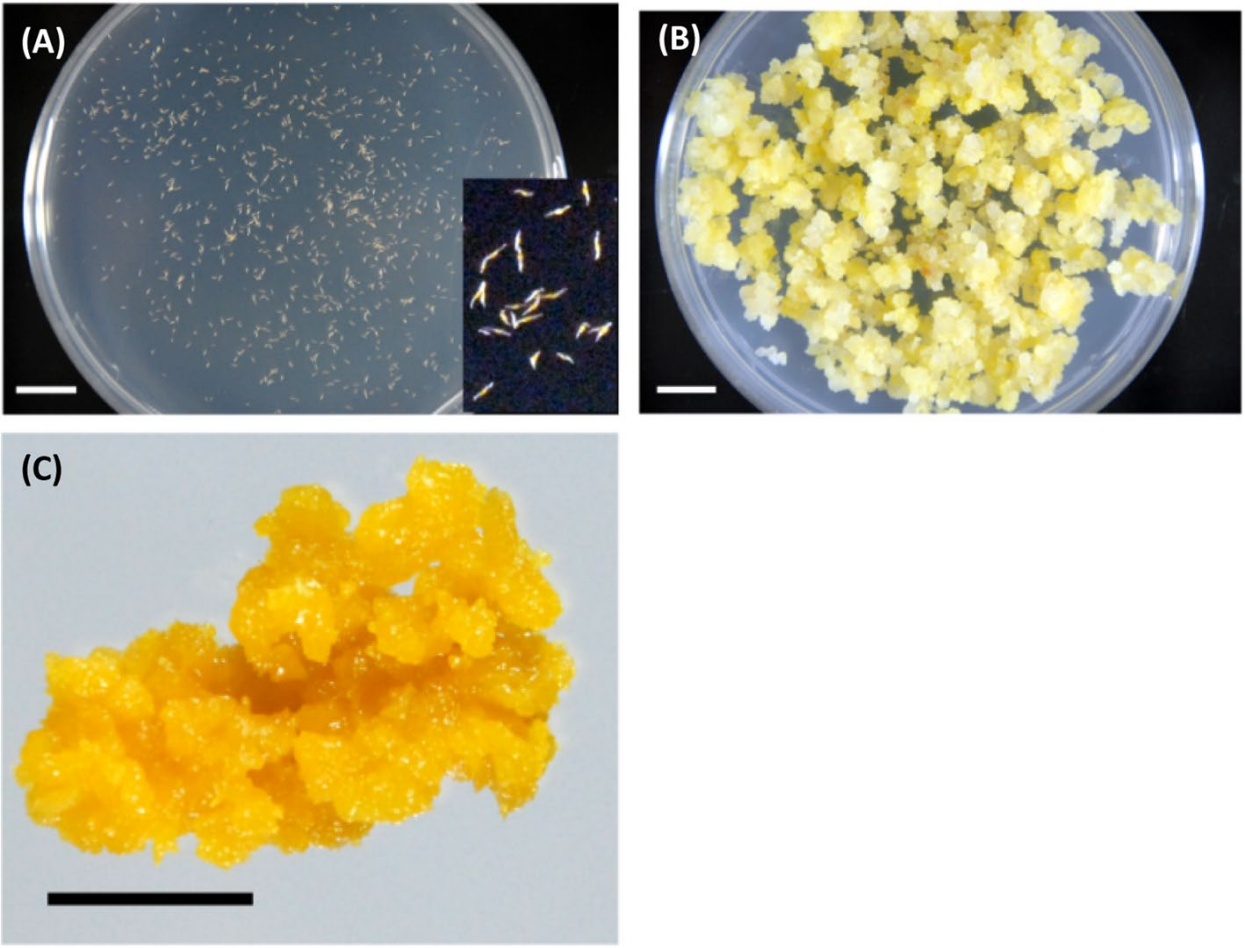
Callus induction, growth, and extraction of *B. formosana*. **A** The seeds of the current year’s flowering were plated for callus induction. **B** Callus with stable growth and loose texture. **C** Appearance of the extract through SFE-CO_2_ from *B. formosana* callus. Scale bar = 1 cm

Plants of *B. formosana* were collected from Taiwan. For callus induction, 2 mg/L 6-BA and 1 mg/L 2,4-D were used. The callus of *B. formosana* was obtained from the seeds of the current year’s flowering with an induction ratio of approximately 90% (Fig. 1A). Subsequently, the callus with good growth and friable texture was selected for the following subculture and went through a long-term subculture, approximately 10 generations, to achieve stable features (Fig. 1B). For extraction, the callus of 10–15 generations was collected and extracted through SFE-CO_2_ after lyophilization for obtaining a pasty extract from *B. formosana* callus (Fig. 1C).

### Cell viability of the B. *formosana* callus extract in dermatological cells

**Fig. 2:**
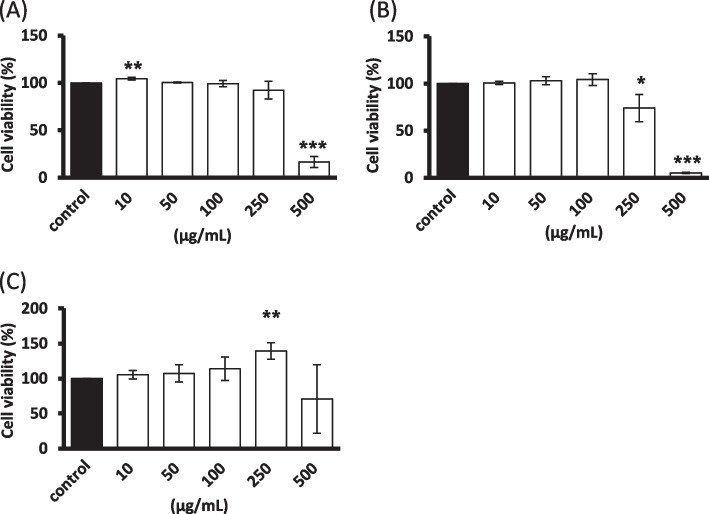
Cell viability in dermatological cells treated with of *B. formosana* callus extract. **A** Hs68 fibroblast cells. **B** HaCaT keratinocytes. **C** B16F10 melanoma cells. Data are presented as means ± SD of triplicate experiments. *p < 0.05, **p < 0.01, and ***p < 0.001 compare with control

For further skin application, the component of *B. formosana* callus extract with low cytotoxicity is necessary. The cell viability of extract on Hs68 fibroblast cells and HaCaT keratinocytes was determined through the MTT method. Figure 2 shows that the callus extract inhibited cell growth unequally in dermatological cell lines after treatment for 24 h at concentrations from 10 to 500 μg/mL. There was no cytotoxicity in Hs68 fibroblast cells at the concentration of 250 μg/mL (Fig. 2A), while lower cell viability with 74.0% was observed in HaCaT keratinocytes (Fig. 2B). Nevertheless, enhanced cell proliferation was observed in B16F10 melanoma cells from 105.4 to 139.2% (Fig. 2C). Inhibition of cell growth was detected in all tested cell lines at the concentration of 500 μg/mL, showing that *B. formosana* callus extract might exhibit cytotoxicity at the concentrations higher than 250 μg/mL.

### Antioxidative activities of B. *formosana* callus extract

#### Intracellular ROS scavenging activity

**Fig. 3:**
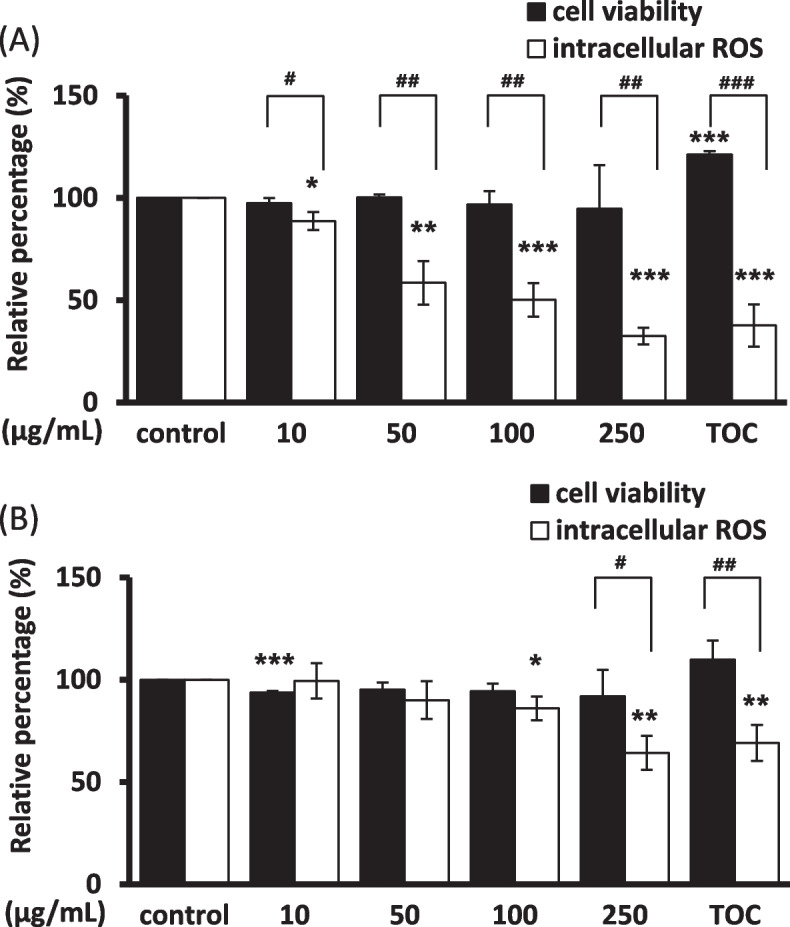
Intracellular reactive oxygen species (ROS) scavenging activity of *B. formosana* callus extract. **A** Relative DCFH-DA fluorescence in Hs68 fibroblast cells. **B** Relative DCFH-DA fluorescence in HaCaT keratinocytes. 0.03% tocopherol was used as the positive control and presented as TOC. Data are presented as means ± SD of triplicate experiments. *p < 0.05, **p < 0.01, and ***p < 0.001 compare with control. ^#^*p* < 0.05, ^##^*p* < 0.01, and ^###^*p* < 0.001 compare with relative cell viability of the same group

The intracellular ROS scavenging effect of *B. formosana* callus extract was investigated with direct H_2_O_2_-induced ROS in Hs68 fibroblast cells and HaCaT keratinocytes using the DCFH-DA assay. The *B. formosana* callus extract exhibited obvious scavenging activity in a dose-dependent manner in fibroblast cells. The relative fluorescence was 88.67 ± 4.35% to 32.50 ± 4.05% at the concentration range of 10–250 μg/mL, compared with the positive control 0.03% of tocopherol was 37.64 ± 10.40% (Fig. 3A). Similarly, keratinocytes exhibited scavenging activity with the relative fluorescence of 86.06 ± 5.72% and 64.30 ± 8.27% at the concentrations of 100 and 250 μg/mL, respectively, compared with the positive control of 0.03% tocopherol was 69.13 ± 8.77% (Fig. 3B). These results indicated that the *B. formosana* callus extract can protect human Hs68 fibroblast cells and HaCaT keratinocytes against H_2_O_2_-induced oxidative stress through the improvement of ROS scavenging activity.

#### Antioxidant genes expression in Hs68 fibroblast cells and HaCaT keratinocytes

**Fig. 4:**
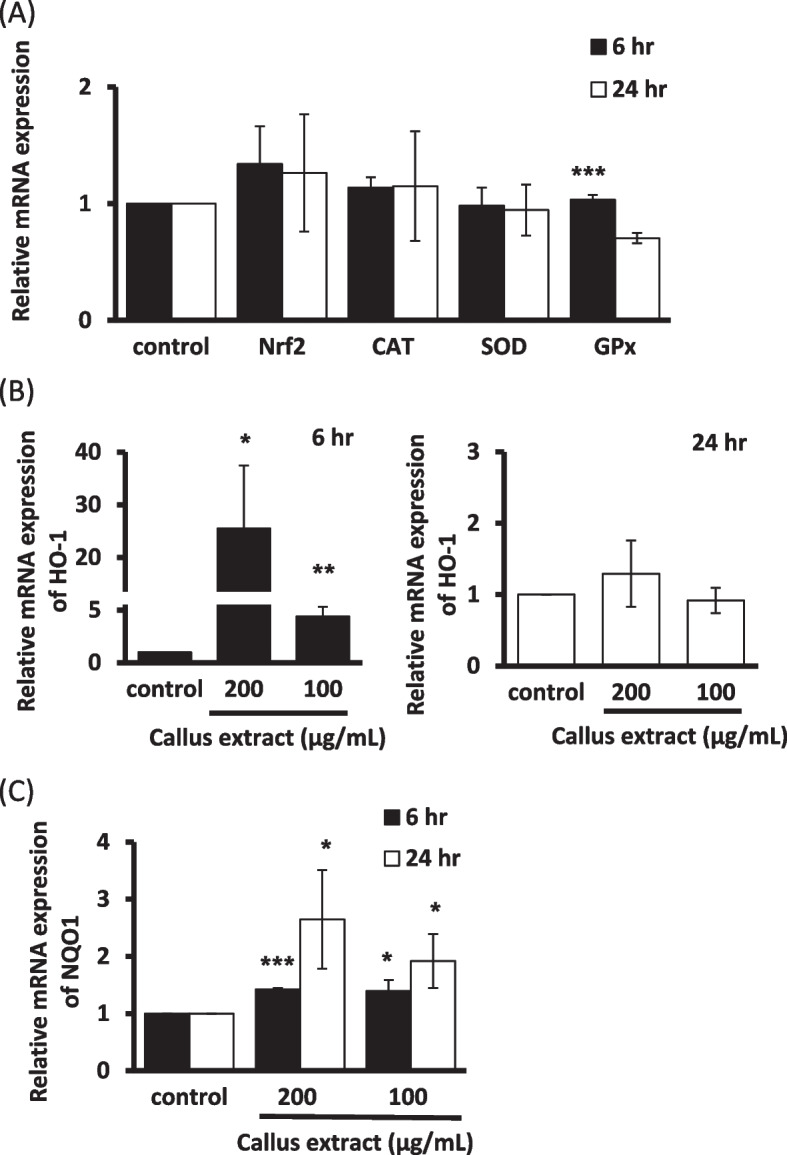
The mRNA expression levels of antioxidant genes were measured in Hs68 fibroblast cells. **A** mRNA expression levels of four antioxidation-related genes, Nrf2, CAT, SOD, and GPx, in cells treated with 200 μg/mL of callus extract for 6 h and 24 h. **B** HO-1 mRNA expression in cells treated with 100 μg/mL and 200 μg/mL for 6 h and 24 h. **C** NQO1 mRNA expression in cells treated with 100 μg/mL and 200 μg/mL for 6 h and 24 h. Data are presented as means ± SD of triplicate experiments. *p < 0.05, **p < 0.01, and ***p < 0.001 compare with control

**Fig. 5:**
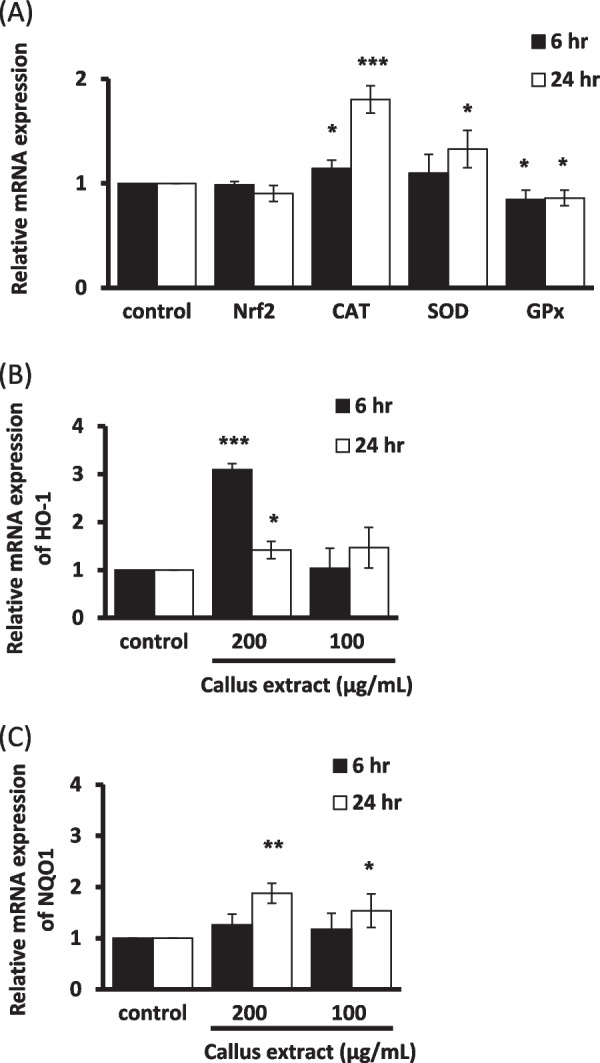
The mRNA expression levels of antioxidant genes were measured in HaCaT keratinocytes. **A** mRNA expression levels of four antioxidation-related genes, Nrf2, CAT, SOD, and GPx, in cells treated with 200 μg/mL of callus extract for 6 h and 24 h. **B** HO-1 mRNA expression in cells treated with 100 μg/mL and 200 μg/mL for 6 h and 24 h. **C** NQO1 mRNA expression in cells treated with 100 μg/mL and 200 μg/mL for 6 h and 24 h. Data are presented as means ± SD of triplicate experiments. *p < 0.05, **p < 0.01, and ***p < 0.001 compare with control

Real-time quantitative PCR analysis was conducted to unveil the candidate antioxidant genes that might contribute to the regulation of *B. formosana* callus extract for the protection of cells against H_2_O_2_-induced oxidative stress. The mRNA expression level of antioxidant genes, including CAT, SOD, GPx, HO-1, NQO1, and the key transcription factor, Nrf2, were evaluated. After treatment with *B. formosana* callus extract for 6 h and 24 h, no expression change was observed in CAT, SOD, GPx, and Nrf2 mRNA levels in fibroblast cells (Fig. 4A). Meanwhile, a remarkable 25-fold increase in the expression level of HO-1 mRNA was detected after 6 h treatment at the concentration of 200 μg/mL (Fig. 4B), though only slight enhancement of NQO1 mRNA expression level was observed after 24-h treatment (Fig. 4C). Similar expression of mRNA levels was observed in CAT, SOD, GPx, Nrf2, and NQO1 genes in keratinocytes (Fig. 5A), while only threefold increase in the expression level of HO-1 mRNA was detected after 6-h (Fig. 5B) treatment. These results indicated that the *B. formosana* callus extract can protect cells against H_2_O_2_-induced oxidative stress through transcriptional regulation.

### Melanogenesis-inhibitory effect

#### Melanogenesis-inhibitory effect in α-MSH-induced B16F10 melanoma cells

**Fig. 6:**
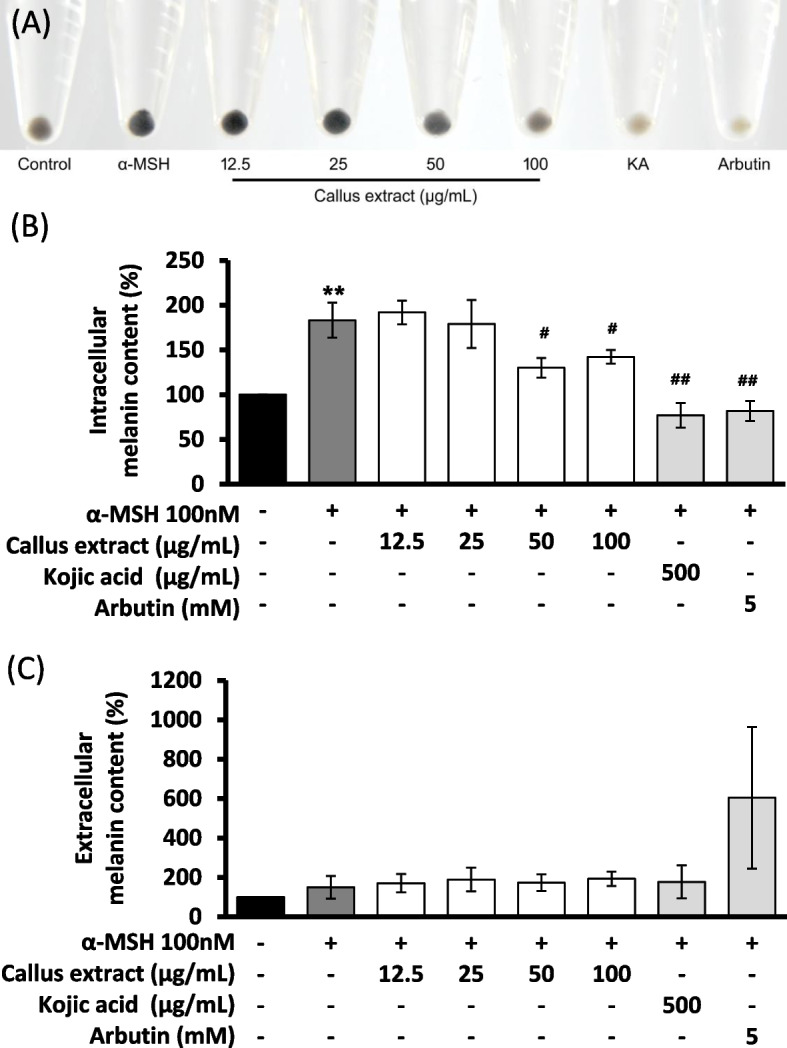
Melanogenesis inhibitory effect of *B. formosana* callus extract in α-MSH-induced B16F10 mouse melanoma cells. **A** Cell pellet collection. **B** Relative intracellular melanin content. **C** Relative extracellular melanin content. The results were normalized with BCA. Data are presented as means ± SD of triplicate experiments. *p < 0.05, **p < 0.01, and ***p < 0.001 compare with control. ^#^ *p* < 0.05, ^##^ *p* < 0.01, and ^###^ *p* < 0.001 compare with α-MSH-treated group

The melanogenesis-inhibitory effect of *B. formosana* callus extract was evaluated by reducing α-MSH stimuli-inducing melanin production on B16F10 mouse melanoma cells. The α-MSH-induced cellular melanin production effectively. The color of the collected cell pellet with α-MSH was dark (Fig. 6A), but faded with the addition of *B. formosana* callus extract in a dose-dependent manner. The α-MSH-induced melanin production in B16F10 melanoma cells was approximately twofold higher than the control group but decreased by adding the positive controls of 500 μg/mL Kojic acid (KA) and 5 mM Arbutin (Fig. 6B). *B. formosana* callus extract decreased α-MSH stimuli-inducing intracellular melanin content in B16F10 melanoma cells by 71.54 ± 10.38% compared to that of the α-MSH-treated group at the concentration of 50 μg/mL (Fig. 6B). Meanwhile, the color of the cell pellet collected from adding *B. formosana* callus extract at the concentration of 100 μg/mL was significantly brighter than that of 50 μg/mL (Fig. 6B). However, no difference in melanin content was detected in the collected media (Fig. 6C).

#### Pigmentation inhibitory effect in zebrafish embryo test

**Fig. 7:**
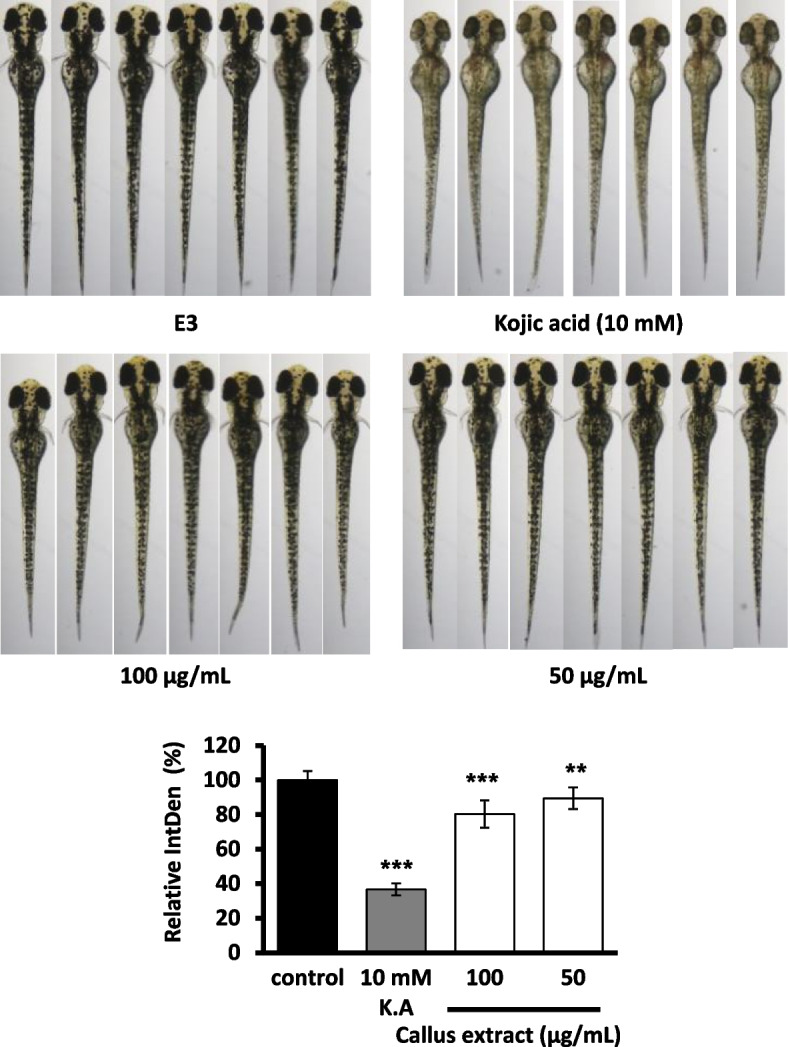
The pigmentation inhibition effect of *B. formosana* callus extract in zebrafish model. The data are expressed as percentage of pigment formation. The results are expressed as means ± SD. ^#^*p* < 0.05, ^##^*p* < 0.01, and ^###^ *p* < 0.001 compare with control

The zebrafish system was employed to investigate the in vivo melanogenesis inhibitory effect and toxicity of *B. formosana* callus extract simultaneously. Morphological abnormality of zebrafish embryo development was not observed in the treatment of *B. formosana* callus extract (Fig. 7). The callus extract exhibited moderate pigmentation inhibition effect with a relative pigment density of 80.27 ± 7.98% and 89.41 ± 6.26% at the concentrations of 100 μg/mL and 50 μg/mL, respectively, compared to that of positive control 10 mM Kojic acid presented a 36.63 ± 3.47% relative pigmentation density (Fig. 7).

## Discussion

Orchidaceae plants commonly difficult to obtain offspring in nature due to the undernourished seed. As Fig. 1, the callus of *B. formosana* could be induced from the seeds and proliferated sustainably, indicating that plant cell culture technology is a feasible strategy for preserving *B. formosana* resources [13]. In addition, a green SFE-CO_2_ technology was used for the extract of *B. formosana* callus. SFE is a relatively eco-friendly and high-efficiency approach compared to traditional solvent or mechanical extraction due to the utilization of supercritical gas fluid, such as CO_2_, a gas that exists in the normal air with a higher diffusion factor to enhance mass transfer [28, 29]. Moreover, CO_2_ molecule reaches supercritical status under the conditions of 31 °C and 74 bar, which can prominently preserve the bioactive compounds in the materials [30]. As mentioned above, the extract of *B. formosana* was obtained from a sustainable resource as well as a green extraction process.

The extract of *B. formosana* callus exhibited low cytotoxicity in fibroblast cells and keratinocytes (Fig. 2). In order to investigate the antioxidative activity of *B. formosana* callus extract, an intracellular ROS scavenging assay was employed. The intracellular ROS scavenging assay presented an obvious effect with 67.5% suppression at 250 μg/mL (Fig. 3), indicating that the *B. formosana* callus extract can protect fibroblast cells and keratinocytes against H_2_O_2_-induced oxidative stress. The antioxidant mechanisms include direct radical scavenging, suppression of ROS formation, and regulation of the antioxidant defense system [14]. SOD, CAT, and GPx are the first-line defense enzymes against ROS [31], and transcription factor Nrf2 mediates the most important signaling pathway against oxidative stress in skin cells [32]. In addition, phase II antioxidant enzymes, such as HO-1 and NQO1, were also employed in the ROS defense system [33]. The Nrf2-regulated HO-1 gene plays a crucial role in the development of oxidative and age-related disorders [34]. NQO1, another Nrf2-regulated gene [35], is one of the two mammalian forms of the NAD(P)H:quinone acceptor oxidoreductases family belonging to obligate two-electron reductase [36, 37]. In this study, the expression levels of SOD, CAT, and GPx, and Nrf2 genes did not change in response to *B. formosana* callus extract treatment after 6 h and 24 h in Hs68 fibroblast cells (Fig. 4A). However, HO-1 mRNA expression levels was over 25-fold higher than that of control in Hs68 fibroblast cells after 6-h treatment as well as NQO1 mRNA expression increased slightly in a dose-dependent manner at 24-h treatment (Fig. 4B-C), indicating the translocation of Nrf2 might be activated and elevated the gene expression of HO-1 and NQO1. Similar gene expression levels of HO-1 and NQO1 were detected in HaCaT keratinocytes (Fig. 5B-C). As a result, the protection mechanism of cells against H_2_O_2_-induced oxidative stress by *B. formosana* callus extract was probably mediated by the induction of antioxidant genes, HO-1 and NQO1, via the activation of Nrf2 translocation. Many Nrf2 activators derived from natural sources have been illustrated. For example, amentoflavone, a biflavonoid found in many plants, potentially protects cells against aging, inflammation, and many diseases by inducing Nrf2 activation [38]. The extracts from plant cells are a valuable source for being abundant antioxidant compounds with anti-aging properties [14].

The melanogenesis-inhibitory effect of *B. formosana* callus extract was determined with melanoma cells (Fig. 6) and zebrafish in vivo assay (Fig. 7). The addition of 50 μg/mL *B. formosana* callus extract decreased 28.46% of α-MSH stimuli-inducing intracellular melanin content in B16F10 cells. Moreover, the addition of 100 μg/mL extract showed a brighter color of the collected cell pellet. In the zebrafish assay, pigment production at the concentrations of both 50 and 100 μg/mL without observable toxicity and the *B. formosana* callus extract effectively suppressed melanogenesis in zebrafish, which was consistent with the result in B16F10 mouse melanoma cells. The results of in vitro and in vivo assays are consistent with the historical dermatology use of *Bletilla* species for skin whitening formulations [2, 39], indicating that *B. formosana* callus possesses similar active ingredients to plants. In addition, a large number of secondary metabolites in *Bletilla* species were identified, such as stilbenes, phenanthrene derivatives, bibenzyls, flavonoids, and phenolic compounds [3]. The previous study has revealed that stilbenoids extracted by SFE are the potential constituents of *Bletilla striata* on melanogenesis-inhibitory activity [40, 41], indicating the *B. formosana* callus extract from SFE might also contain similar constituents and contribute to the melanogenesis-inhibitory activity. Though the secondary metabolites profile and content in plant cells might not be the same as those of plants, there are still some cases exhibiting more content of bioactive compounds in cells compared to plants, such as shikonin in the cell culture of *Lithospermum erythrorhizon* [42] and berberine in the cell culture of *Berberis vulgaris* [43], showing the potential of plant cell cultures as a sustainable alternative of cosmetic ingredients.

## Conclusions

In conclusion, *B. formosana* callus extract presented antioxidative activity and melanogenesis-inhibitory properties without significant effect on cell growth inhibition in either HaCaT keratinocytes or Hs68 fibroblast cells. *B. formosana* callus extract protected cells against H_2_O_2_-induced oxidative stress probably through the activation of Nrf2/HO-1 pathways. Additionally, *B. formosana* callus extract exhibited a melanogenesis-inhibitory effect consistent with the traditional utilization of *Bletilla* species, indicating the utilization potential of *B. formosana* callus as a replacement resource from the endangered plant.

These findings suggest that *B. formosana* callus extract produced through a sustainable and eco-friendly process presents a promising utilization as a functional material for dermatology treatments. However, the underlying molecular mechanisms of *B. formosana* callus extract need further studies.

## Patents

Ruei-Ting Wang, Yi-Cian Lai, and Wei-Ping Wang. Dedifferentiated cell extract of *Bletilla* and its applying and cosmetic products containing the same. Patent TW I767559, 9 February 2019.

## Data Availability

All data generated or analyzed during this study are included in this published article.

## Abbreviations

TCM: Traditional Chinese Medicine
SFE-CO_2_: Supercritical fluid CO_2_ extraction
ROS: Reactive oxygen species
NaOCl: Sodium hypochlorite
6-BA: 6-Benzylaminopurine
2,4-D: 2,4-Dichlorophenoxyacetic acid
DMSO: Dimethyl sulfoxide
DMEM: Dulbecco’s modified Eagle medium
MTT: 3-(4,5-Dimethylthiazol-2-yl)-2,5-diphenyltetrazolium bromide
DCFH-DA: 2′,7′-Dichlorofluorescin diacetate
Nrf2: Nuclear factor erythroid 2-related factor 2
CAT: Catalase
SOD: Superoxide dismutase
GPx: Glutathione peroxidase
HO-1: Heme oxygenase-1
NQO1: NAD(P)H quinone oxidoreductase-1
GAPDH: Glyceraldehyde-3-phosphate dehydrogenase
α-MSH: α-Melanocyte-stimulating hormone
MC1R: Melanocortin 1 receptor
NaOH: Sodium hydroxide
BCA: Bicinchoninic acid
hpf: Hours post-fertilization
